# Suspected cluster of *Neisseria meningitidis* W invasive disease in an elderly care home: do new laboratory methods aid public health action? United Kingdom, 2015

**DOI:** 10.2807/1560-7917.ES.2019.24.23.1900070

**Published:** 2019-06-06

**Authors:** Jonathan Lawler, Jay Lucidarme, Sydel Parikh, Lorna Smith, Helen Campbell, Ray Borrow, Steve Gray, Kirsty Foster, Shamez Ladhani

**Affiliations:** 1 Public Health England North East, Newcastle upon Tyne, United Kingdom; 2 Public Health England Meningococcal Reference Unit, Manchester, United Kingdom; 3 Public Health England National Infection Service, London, United Kingdom

**Keywords:** Neisseria meningitidis serogroup W-135, multilocus sequence typing, meningococcal infections, pneumonia, disease outbreaks, whole genome sequencing, elderly, care home

## Abstract

In 2015, a suspected cluster of two invasive meningococcal disease (IMD) cases of serogroup W *Neisseria meningitidis* (MenW) occurred in elderly care home residents in England over 7 months; case investigations followed United Kingdom guidance. An incident control team reviewed epidemiological information. Phenotyping of case specimens informed public health action, including vaccination and throat swabs to assess carriage. Whole genome sequencing (WGS) was conducted on case and carrier isolates. Conventional phenotyping did not exclude a microbiological link between cases (case 1 W:2a:P1.5,2 and case 2 W:2a:NT). After the second case, 33/40 residents and 13/32 staff were vaccinated and 19/40 residents and 13/32 staff submitted throat swabs. Two MenW carriers and two MenC carriers were detected. WGS showed that MenW case and carrier isolates were closely related and possibly constituted a locally circulating strain. Meningococcal carriage, transmission dynamics and influence of care settings on IMD in older adults are poorly understood. WGS analyses performed following public health action helped to confirm the close relatedness of the case and circulating isolates despite phenotypic differences and supported actions taken. WGS was not sufficiently timely to guide public health practice.

## Background

*Neisseria meningitidis* (Nm) is a major cause of meningitis and septicaemia globally and is associated with significant mortality and long-term morbidity among survivors. The epidemiology of invasive meningococcal disease (IMD) is evolving and geographical and temporal trends change over time [1]. In England, IMD occurs most frequently in children under the age of 5 years with the highest incidence in infants aged less than 1 year [2]. In Europe and other industrialised countries, the incidence of serogroup B *N.* *meningitidis* (MenB) has declined but remains a major cause of IMD [1]. In England, during the epidemiological year 2014/15 (July–June, intended to capture winter peak of disease), MenB accounted for 58% (418/724) of laboratory-confirmed IMD (all ages) and 74% (240/289) of laboratory-confirmed IMD in children less than 5 years old [2].

In contrast, meningococcal groups W (MenW) and Y (MenY) have historically been relatively uncommon causes of IMD, occurring more frequently in adults aged over 65 years who usually have underlying comorbidities. These capsular serogroups often have different clinical presentations from MenB, including pneumonia, septic arthritis, endocarditis and epiglottitis/supraglottitis [3]. In such cases, IMD may not be considered in the differential diagnosis and is only confirmed when *N. meningitidis* is unexpectedly isolated from blood culture or from culture of other sterile sites.

**Figure 1:**
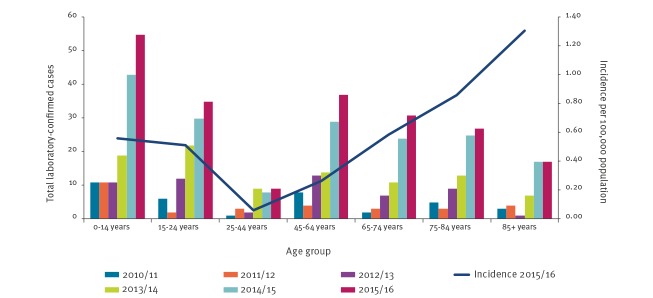
Distribution of laboratory-confirmed MenW cases by age group and epidemiological year, England, 2010–2015 (n = 603)

Although the overall incidence of laboratory-confirmed IMD in England has declined from 1,438 cases (3.8/100,000 population based on midyear population estimates [4]) in 2001/02 to 811 cases (1.5/100,000 population) in 2015/16, the incidence of MenW IMD has increased every year from 2009 during this period [5]. In England, MenW cases nearly quadrupled from 55 in 2012/13 to 211 in 2015/16 (Figure 1) when the highest incidence was in those aged under 5 years, followed by those over 65 years of age [2]. MenW cases in the latter group increased fourfold from 17 (0.2/100,000 population) in 2012/13 to 75 (0.8/100,000 population) in 2015/16; the highest incidence in this age group was in those aged 85 years and older (1.3/100,000 in 2015/16) although case numbers were small. In 2012/13, all age case fatality ratio (CFR) in England was 13% for MenW IMD with the highest CFR in those aged 65 years or older (22%) [6].

MenW: serogroup W *Neisseria meningitidis*.

Source: Public Health England surveillance data [2].

The strain responsible for the increase in MenW IMD belongs to the sequence type (ST-)11 clonal complex (cc11) which is associated with multiple serogroups, in particular MenC and MenW, and has a tendency to cause outbreaks of relatively severe disease [7-9]. By 2013/14, strains belonging to cc11 accounted for nearly all MenW cases in individuals aged 5–64 years and two-thirds of cases in those aged less than 5 years and 65 years and older [6]. The vast majority of MenW cc11 strains are indistinguishable by conventional typing schemes (PorB serotype 2a and PorA serosubtype P1.5,2) and belong to ST-11, as defined by seven-locus multilocus sequence typing (MLST) [10]. High-resolution core-genome MLST (cgMLST; based on ca 1,600 core genes), has recently revealed that cc11 comprises two main lineages, lineage 11.1 (including all major W:cc11 strains and some serogroup B and C strains) and lineage 11.2 (predominantly serogroup B and C strains). It also revealed considerable diversity among MenW cc11 and demonstrated that the expanding United Kingdom (UK) strain belonged to the so-called South American-strain sublineage having descended from a strain that emerged in South America in the early 2000s [11,12]. Subsequently, a descendant of the ‘original UK strain’ emerged in 2013 (the ‘2013-strain’) and has since expanded and spread to several European countries, Australia and Canada [13-15].

Laboratory confirmation of IMD and characterisation of the meningococcal strain responsible has important public health implications, as there is a need to offer timely antibiotic chemoprophylaxis and vaccination (depending on the serogroup). In addition to monitoring cases for national surveillance, diagnosing IMD across all age groups is important to rapidly identify and manage clusters and outbreaks. Most reported IMD outbreaks and clusters have involved children and young adults (aged 0–25 years) and occurred in institutional settings including school and pre-school settings, universities and the military [16]. There are limited published reports of IMD clusters or outbreaks involving those aged over 25 years or outside these settings.

‘Extended’ MLST such as cgMLST provides considerable additional resolving power for cluster and outbreak investigations where routine (such as 7 gene) schemes prove to be non-discriminatory [17]. The Meningitis Research Foundation Meningococcus Genome Library (MRF-MGL) [18] facilitates these analyses by providing online open-access to annotated draft genomes for all English, Welsh and Northern Irish case isolates received by the Public Health England (PHE) Meningococcal Reference Unit (MRU) since July 2010, alongside tools for their analysis.

### Detection of suspected cluster of MenW IMD and initial public health response

In 2015, two cases of MenW IMD occurred in residents over 85 years old at a 46-bed elderly care home in North East England over a 7-month period; both cases had single bedrooms. According to national PHE guidance for the public health management of meningococcal disease, staff and other residents in nursing/residential homes are not routinely defined as close contacts following a single case of IMD [16]. Therefore, chemoprophylaxis was not provided to staff or residents during the initial public health management of either case.

The prolonged interval (7 months) between the two cases raised questions about whether the infections were related and whether action was required to minimise the risk of further cases in residents and staff. Here, we describe the case investigations, carriage data and use of whole genome sequencing (WGS) to inform public health action.

## Methods

### Laboratory investigations

Serogrouping, serotyping and serosubtyping were performed as previously described [19]. Paired-end genome sequence analysis was performed using the Nextera XT library preparation kit (Illumina, Inc. San Diego, California) on the Illumina HiSeq 2500 (100bp read lengths; case isolates) or MiSeq (300bp read lengths; carrier isolates) platform. DNA input was 1ng of genomic DNA per isolate. The reads were assembled using Velvet (version 1.2.08) [20] in conjunction with Velvet optimiser (version v2.2.4) [21]. Genome assemblies were deposited on the PubMLST *Neisseria* database [22,23] and MRF-MGL where they underwent automated annotation at > 2,000 genomic loci including the seven MLST loci and 1,600 core genome loci. The isolates were compared with all remaining W:cc11 isolates on the MRF-MGL (n = 387; accessed 24 Feb 2016) and an evolutionarily-distant B:cc11 lineage 11.2 reference strain (PubMLST ID 21361) in terms of 1,546 core genome loci using the PubMLST genome comparator tool. Incomplete loci (e.g. those that extend beyond the end of a contig) were ignored on a pairwise basis and paralogous loci were excluded from the analysis [6,11]. The resulting distance matrix (counting the number of different alleles, per pair of isolates, among the 1,546 loci compared) was visualised as a Neighbor-net network using SplitsTree4 (version 4.12.8).

### Cluster investigation

Case 1 was admitted to hospital with acute respiratory distress and fever (temperature 41 °C). Bacteraemic pneumonia was diagnosed and the case died 6 days after onset of illness. *N. meningitidis* was isolated from blood cultures collected on admission and further typing data confirming *N. meningitidis* serogroup W, PorB serotype 2a and PorA serosubtype (P1.5,2) infection were received from the PHE MRU. 

Case 2 was admitted to hospital with acute onset of fever, tachycardia and hypotension and was treated for respiratory sepsis unsuccessfully. The case died 7 days after onset of illness, which was 7 months after case 1. *N. meningitidis* was isolated from blood cultures collected on admission and further typing confirming *N. meningitidis* serogroup W, PorB serotype 2a and PorA serosubtype (NT; non-typeable) was received from the MRU. 

Following identification of MenW IMD in case 2, an assessment was undertaken to determine whether the cases could be linked and whether they might be part of a cluster.

### Case definition

The case definition, based on PHE guidance [16], was a resident or member of staff with a clinical diagnosis of meningitis, septicaemia or other invasive disease and MenW detected by culture or molecular methods in a normally sterile site. Current UK guidance defines IMD clusters in educational settings as two or more confirmed cases of IMD with the same capsular group occurring within a 4-week period [16]. Although the temporal criterion of the cluster definition was not met in this situation, the occurrence of two cases of MenW IMD in residents of the same care home was extremely unusual. Given the limited knowledge of meningococcal carriage in the elderly and IMD clusters within residential care settings, an incident control team (ICT) with representatives from the PHE North East Health Protection Team, the national PHE Immunisation team at the Centre for Infectious Disease Surveillance and Control (CIDSC) and PHE MRU was established to consider further action.

### Epidemiological investigation

The care home provides care for a maximum of 46 residents (aged 65 years or older) including those with dementia-related conditions. At the time of investigation there were 40 residents (median age 83 years, range 66–98 years). Bedrooms are located over three floors and each resident has a single bedroom with en suite toilet. There are shared communal lounges on each floor; residents on the ground and first floors use the ground floor dining room. Accommodation and care for those with moderate to severe dementia-related conditions are provided in a separate self-contained unit on the second floor. Residents in the dementia unit do not have regular contact with those in other units, but the staff work between all parts of the care home.

Case 1 and case 2 had been resident in the care home for more than a year before the first onset of illness. Case 1 had been resident in the separate dementia unit for 11 months before onset, while case 2 was resident on the first floor. Both cases had been resident on the same floor for a 2-week period 11 months before the onset in case 1. However, the cases had no contact with each other during this period nor for the remainder of their residencies spent on separate floors. The two cases shared bathrooms and communal dining areas with other residents on their respective floors and engaged in social activities during the day. Staff (median age 43 years, range 20–77 years) worked across all floors of the home and would have had contact with both cases.

The ICT agreed that given the interval between onsets these could be sporadic unlinked infections. However, available phenotyping did not provide sufficient information that isolates were different and the ICT was, therefore unable to determine that the cases were not microbiologically linked.

### Control measures

Consequently, based on the case fatalities and uncertainty about possible on-going carriage and transmission in the care home, the ICT decided that MenW vaccination should be offered to all residents and staff to protect against MenW disease. As the rationale for vaccination was based on the possibility of persisting carriage within the care home, the ICT made the pragmatic decision that vaccination would be offered to all those who were resident or employed within the care home at the time of planned vaccination.

The ICT also agreed that the actions in the care home offered the opportunity to further the understanding of carriage in this setting and so agreed that throat swabs for *N. meningitidis* culture would be collected from residents and staff at the same time that vaccination was given. Vaccination and swabbing took place at the care home ca 12 weeks after the diagnosis of case 2. Arrangements were also made so that staff could obtain vaccination from their family doctor if they were unable to attend the session.

## Results

### Vaccination and swabbing uptake

A single dose of MenACWY conjugate vaccine (Nimenrix®; Pfizer Inc) was offered to 40 residents and 32 staff; uptake was 83% (33/40) in residents and 78% (25/32) in staff. Throat swabs were collected from 48% (19/40) of residents and 40% (13/32) of staff. Of the 19/32 staff who did not submit throat swabs, one declined and the remainder did not attend the session.

### Laboratory investigations

MenW was isolated in throat swabs from one resident in their 80s and one care worker in their 20s, while MenC was isolated from two care workers (Table). All isolates (two case isolates and four carrier isolates) underwent genome sequence analysis. The two MenC carrier isolates were identified as having PorA P1.5–1,2–2 and belonging to ST-1157 (cc1157).

**Table:** Meningococcal case and carriage isolates including serogroup, phenotype and clonal complex, England, 2015 (n = 6)

IMD case/carriage	Month of specimen collection^a^, 2015	Staff/resident	Age group (years)	Serogroup	Phenotype		Molecular type
					*PorA*	*PorB*	Sequence type (clonal complex)
Case 1	0	Resident	80–90	W	2a	(P1.5/P1.2/NT)	ST-11 (cc11)
Case 2	7	Resident	90+	W	2a	(NT/NT/NT)	ST-11 (cc11)
Carriage	10	Resident	80–90	W	2a	(P1.5 / P1.2 / NT)	ST-11 (cc11)
Carriage	10	Staff	20–30	W	2a	(P1.5 / P1.2 / NT)	ST-11 (cc11)
Carriage	10	Staff	20–30	C	NT	(P1.5 / P1.2 / NT)	ST-1157 (cc1157)
Carriage	10	Staff	30–40	C	NT	(P1.5 / P1.2 / NT)	ST-1157 (cc1157)

Cc: clonal complex; IMD: invasive meningococcal disease; NT: not typable; ST: sequence type.

^a^ Month of specimen collection from case 1 was set to 0. The following months indicate the number of months following the specimen collection from case 1.

**Figure 2:**
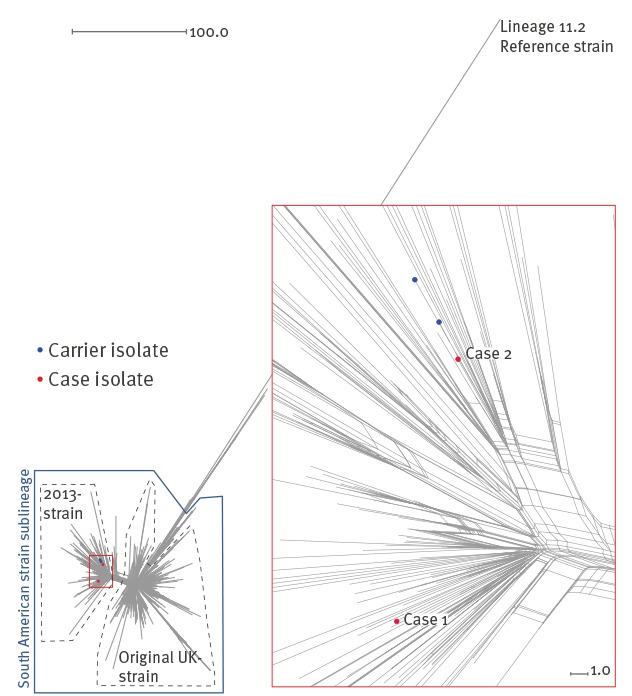
Neighbor-net network of care home case and carrier isolates and MenW cc11 isolates from England, Wales and Northern Ireland, July 2010–December 2015

All four of the MenW isolates (two cases and two carriers) belonged to the UK-2013 strain of the South American strain sublineage (Figure 2). The Neighbor-net network exclusively placed the isolate from case 2 and the two carrier isolates into a distinct sublineage differing from one another at between three and six loci (among the 1,546 compared). The isolate from case 1 exclusively formed a distinct sublineage and differed from the other three isolates at between 12 and 19 loci. For comparison, the case 1 isolate differed from the other 387 isolates by between 11 and 239 loci (mean 51 loci, median 52 loci, SD 27 loci). Of the 12 isolates that differed from the case 1 isolate at < 15 loci, six were isolated before the onset of case 1’s symptoms (isolates from 2014-15) and six afterwards (all during 2015). These 12 isolates were widely distributed across England and Wales. The reported differences between the four case and carrier isolates were further evaluated by performing BLAST searches and/or nt alignments of the corresponding alleles/isolates. Several reported differences were a result of an identical allele being assigned a full-length variant ID in one isolate and a truncated allele ID in the other (despite possessing the full-length allele). In addition, several genes were erroneously classified as ‘missing’ due to fragmented assembly. After excluding erroneous differences, the two case isolates were found to differ at two loci while the case and carrier isolates collectively differed at between one and six loci.

Cc: clonal complex; MenW: serogroup W *Neisseria meningitidis*; ST: sequence type.

Neighbor-net phylogenetic network based on a comparison of 1,546 core genes among all serogroup W, ST-11 clonal complex genomes on the PubMLST database, including two care home case isolates (n = 387; accessed 24 Feb 2016), and two care home carrier isolates. The case and carrier isolates all belonged to the 2013 strain of the South American strain sublineage. The scale bar indicates the number of differences among the 1,546 loci compared.

## Discussion

We describe two cases of MenW IMD in elderly residents of a care home who presented with bacteraemic pneumonia due to a relatively rare but expanding hypervirulent clone. In the UK IMD is infrequently diagnosed in care home residents and two cases from the same home was unusual. Although the increase in MenW IMD in the UK was initially seen in adults aged 65 years and older [3], we believe that IMD may be underdiagnosed in this age group who frequently present with pneumonia and symptoms other than meningitis. *Streptococcus pneumoniae* is the most common pathogen associated with community-acquired pneumonia (CAP) [24] and consequently there may be a low index of suspicion for IMD in adults aged 65 years and older with pneumonia.

Current UK guidelines for the management of CAP (that does not require hospitalisation) advise empirical treatment with oral antibiotics; a microbiological investigation is not required [25]. Consequently, some patients may be treated for suspected CAP before being admitted to hospital, which could affect the sensitivity of subsequent microbiological investigations to detect *N. meningitidis* and other pathogens. Meningococcal PCR testing is widely available in the UK, but it is not routinely conducted for CAP [25] and must be requested if IMD is clinically suspected [16].

The highest rates of meningococcal carriage have typically been reported in teenagers (15–19 years) and young adults (20–24 years) [26], but it is not known whether carriage in these age groups is responsible for transmission to older adults (65 years and older). It is also not known whether IMD in older adults occurs predominantly in those living in extended families i.e. exposure to children and teenagers (aged 0–19 years), or in institutions such as care homes, where transmission dynamics may be different. The cases we report here support this lack of information about the transmission dynamics and risk of IMD in older adults as well as the influence of particular settings, such as residential care homes, on these factors.

We found carriage of two meningococcal serogroups in staff (MenW cc11 and MenC cc1157). The detection of MenC carriers was an unexpected finding, but cc1157 carriage appears relatively common in the UK and has been reported among university students [27,28], including a multicentre study where cc1157 accounted for at least 449/8,462 (5.3%) of UK carrier isolates collected from 1999 to 2001 [28]. However, cc1157 is not frequently associated with invasive disease; within the MRF-MGL (accessed 20 Mar 2019) cc1157 accounted for 21/3,659 (0.6%) invasive disease cases in England, Wales and Northern Ireland, none of which were MenC or had PorA P1.5–1,2–2 [28]. Consequently, cc1157 carriage was not of particular clinical concern. Community MenC carriage in adults is not well-described as most carriage studies involve younger cohorts and preceded the introduction of the MenC vaccine. The role of meningococcal carriage in staff as a potential reservoir for transmission of infection to residents in long-term elderly care settings is not well understood and the relevance of this finding is, therefore, unclear.

The 7-month interval between onsets and the difference in the PorA phenotype helped answer the question of whether these cases occurred due to the persistent communal carriage of the same strain or unrelated sporadic infections. WGS showed the case 2 and MenW carrier isolates to be very similar, suggesting possible on-going circulation of the strain responsible for the cases at the time of swabbing and supporting the public health actions taken. Given the national circulation of similarly close strains, however, the possibility of separate introductions of the corresponding isolates could not be ruled out.

Close prolonged contact is required for transmission of meningococci [16,26,29] and secondary cases usually occur within 1 week of the primary case [30]. The UK guidance recommends that close contacts of index cases with suspected IMD are offered antibiotic chemoprophylaxis which provides highly effective short-term protection against secondary cases of all serogroups [16]. In the UK, MenC and MenB are included in the routine infant vaccination schedule, introduced in 1999 and 2015, respectively [31]. MenC vaccine was offered to 13–14-year-olds and new university entrants aged ≤ 25 years until 2015 when, in response to the national MenW outbreak, it was replaced with the MenACWY conjugate vaccine for these age groups alongside a national outbreak control programme offering the vaccine to all 14-18 year olds and new university entrants [32]. In the UK, MenACWY conjugate vaccine is also offered to close contacts of confirmed cases of serogroup A, C, W or Y infection unless vaccinated within the preceding 12 months [32].

According to UK guidance at the time of the cluster [16], care home residents and staff were not routinely considered to be household-type contacts and this has been retained in updated guidance [32]. During the investigation, the question arose as to whether this advice should be reviewed. The limited evidence about transmission in residential care facilities for the elderly may reflect under-diagnosis and/or under-reporting in this age group. It may also be indicative of a genuinely low incidence of secondary transmission. In England, enhanced surveillance of IMD now includes questions about care home residence and prospective studies of possible transmission in this setting may be possible.

In our public health management, routine phenotyping did not exclude a microbiological link between cases while WGS, in conjunction with carriage screening, suggested possible wider circulation of the strain responsible for the second case. WGS is not yet sufficiently timely to guide public health practice on its own and public health specialists must continue to act on the information available at the time, recognising that initial risk assessments may change in light of information that becomes available at a later stage. This situation is not unique to the management of IMD or to the use of WGS.

The incidence of IMD caused by MenW:cc11 has increased in several European countries in recent years, with the highest number of cases in those aged ≥ 65 years [33]. The lack of understanding about meningococcal transmission dynamics and possible under-diagnosis of IMD in this age group and the management of cases in care settings are unlikely to be unique to the UK. This highlights the importance of ongoing European surveillance and sharing practical experience of using new laboratory techniques in public health practice.
